# Toxicity Prediction Method Based on Multi-Channel Convolutional Neural Network

**DOI:** 10.3390/molecules24183383

**Published:** 2019-09-17

**Authors:** Qing Yuan, Zhiqiang Wei, Xu Guan, Mingjian Jiang, Shuang Wang, Shugang Zhang, Zhen Li

**Affiliations:** College of Information Science and Engineering, Ocean University of China, Qingdao 266100, China; yuanqing@stu.ouc.edu.cn (Q.Y.); weizhiqiangouc@163.com (Z.W.); guanxu@stu.ouc.edu.cn (X.G.); jmj@stu.ouc.edu.cn (M.J.); ws@stu.ouc.edu.cn (S.W.); zhangshugang@hotmail.com (S.Z.)

**Keywords:** deep learning, Tox21, toxicity prediction, convolutional neural networks

## Abstract

Molecular toxicity prediction is one of the key studies in drug design. In this paper, a deep learning network based on a two-dimension grid of molecules is proposed to predict toxicity. At first, the van der Waals force and hydrogen bond were calculated according to different descriptors of molecules, and multi-channel grids were generated, which could discover more detail and helpful molecular information for toxicity prediction. The generated grids were fed into a convolutional neural network to obtain the result. A Tox21 dataset was used for the evaluation. This dataset contains more than 12,000 molecules. It can be seen from the experiment that the proposed method performs better compared to other traditional deep learning and machine learning methods.

## 1. Introduction

Our daily lives are exposed to a large number of compounds including the environment, foods, skin care products and medicines. In order to protect human health and the human body from potentially harmful substances, these compounds must pass reliable adverse reaction tests, especially toxicity tests. In addition, the actual chemical experiments could test the toxicity of compounds, but they consume considerable manpower, financial and material resources, and many experiments are very time-consuming and may be involved in ethical issues. With the rapid development of artificial intelligence, computers are used to accomplish these tasks. This is a powerful tool for compound property prediction. However, the accuracy of the computer-aided method is still a problem which needs to be solved.

From the end of the last century to the present, as machine learning continues to flourish in various fields, its application in biomedicine has become more widespread [1]. Further, artificial intelligence has been widely used in drug design [2]. For example, in terms of the binding between proteins and small molecules, an energy-based method for the prediction of protein-ligand binding sites was proposed [3]. Michael S. Lee et al. [4] researched the calculation of the binding affinity through the path and endpoint approaches. George E. Dahl et al. [5] proposed multitask neural networks for quantitative structure–activity relationship (QSAR) predictions. 

For the QSAR predictions including toxicity predictions, the molecular feature and fingerprints are very important for the performance of the prediction. The simplified molecular input line entry system (SMILES) [6] is a popular language used to represent molecules. Some methods focused on the 2D structure of the molecule and others focused on the 3D information collection. For example, the Barnard Chemical Information Ltd. (BCI) fingerprint is generated according to six different families, including augmented atoms, atom/bond sequences, atom pairs, ring composition fragments, ring fusion fragments and ring ortho fragments [7]. As a molecule is a graph, which means the connection between each atom could provide useful information, there are some methods researching on how to utilize graph information. For example, the extended-connectivity circular fingerprints (ECFP) [8] is the standard circular fingerprints based on the Morgan algorithm [9]. It selects each heavy atom as the start node and generates the bag of fragments iteratively. Each fragment is hashed into a representation with a fixed-length. The ECFP could be calculated very rapidly and the information of substructures collected. Furthermore, in order utilize the 3D information, Mahendra Awale et al. [10] proposed a fingerprint combining the molecular shape extracted from the 3D-structures and the 2D atom pair fingerprint.

All these features could be fed into certain machine learning methods for prediction. C.Y. Zhao [11] used a support vector machine (SVM) for toxic activity prediction, and compared it with two other methods, the multiple linear regression (MLR) and the radical basis function neural network (RBFNN). It was demonstrated that the SVM performed best among these three methods of different data sets. Furthermore, Hui Zhang et al. [12] proposed a GA-CG-SVM method which utilized the genetic algorithm (GA) to select the features and GA to optimize the parameters. A k-nearest neighbor (kNN) model was introduced for the prediction of acute contact toxicity of pesticides [13]. Decision trees were utilized to estimate sensory irritation potency of volatile organic chemicals [14]. In addition, random forest (RF) [15], which combines multiple decision trees, was also used for toxicity prediction. Renee Solimeo et al. [16] combined the kNN method and RF method together to predict chemical ocular toxicity. On the other hand, some generative machine learning models have also been used in toxicity prediction. The naïve Bayes model was utilized for predicting the carcinogenicity of chemicals and provided guidance for drug design [17]. The linear discriminant analysis (LDA) was also introduced to predict idiosyncratic hepatotoxicity [18]. However, because traditional machine learning methods are based on hand-craft features mentioned before, it is often difficult to find important attributes and positional information that truly affect the toxicity of the molecule, which may adversely affect the results. 

With the development of the deep learning method, it plays a significant role in the research fields of biology and medicine. For example, recurrent neural networks (RNN) were utilized to generate focused molecule libraries [19], and a deep neural network with a multi-task learning setting was used for a binding prediction [20]. Moreover, Mohammed AlQuraishi et al. [21] also proposed the end-to-end differentiable learning method to predict the protein structure. In the prediction of molecular activity, a structure-based convolutional neural network was proposed to predict the biological activity of small molecules in drug discovery applications, known as AtomNet [22]. In recent years, the research on the toxicity prediction of deep learning on molecules has been continuously proposed. With the help of deep learning, the features could be extracted automatically. Youjun Xu et al. [23] proposed a regression model and multiclass models for toxicity prediction with the assistance of automatic chemical feature extraction and deep learning. Kedi Wu et al. [24] used topology and multitask deep neural networks for quantitative toxicity prediction. Swamidass et al. [25] designed a 4-layer deep neural network (DNN) to predict sites of epoxidation, which provides atomic-level and molecule-level precise information based on 702 expoxidation reactions. Abdul Karim et al. [26] combined the strings, images and numerical features together, and proposed a multimodal deep learning method for toxicity prediction. 

For the deep learning method, the dataset selection is curial to evaluate the performance of the method. The IGC50 dataset [24] is a popular dataset which records acute aquatic toxicity of the compound on the tetrahymena pyriformis population. It contains 1792 compounds which are stored in SMILEs format. The fully convolutional neural network (FCNN), the convolutional neural network (CNN), RNN and the combination of these methods were implemented to test the performance of the IGC50 dataset [26]. Another dataset for the prediction of toxicity is the dataset of drug-induced liver injury [27] which contains 1087 compounds for training and 120 compounds for testing. Furthermore, an RNN-based method has been implemented on this dataset for toxicity prediction [28]. The ToxCast dataset collects toxicology data through vitro high-throughput screening, which covers 600 experiments on 8615 compounds. For each assay, only 205 molecules on average could be used for training and testing [29]. The Tox 21 [30] is the most famous dataset used for the prediction of toxicity. It contains more than 12,000 compounds with 12 properties. With the assistance of this dataset, this study could evaluate the method with respect to 12 properties, which could demonstrate the generalization of the method. There are also many deep learning methods performed on the Tox21 dataset, such as CNN [31], the pre-trained autoencoder [32], the associative neural networks (ASNN) [33] and DNN [34], which could be used for a horizontal comparison. Therefore, the Tox21 dataset was selected as the evaluation dataset in this paper.

Although there are some methods focusing on toxicity prediction using deep learning methods, most of them have utilized the SIMLEs string as the input of the network ignoring the spatial information of the molecule, which is an important factor of molecular activity. At the same time, the grid method has been utilized to extract the characteristics of the molecule in different grid locations, which could introduce the spatial information to the molecular feature. For example, K_DEEP_ [35] constructed a multi-channel three-dimensional grid for characterizing the binding sites of proteins and small molecules, a Van der Waals force-like method was used to calculate the contribution of all molecules to each voxel of grid within a certain range, and the grid was fed into a deep neural network to predict the location of the binding site. Although the grid method provides a novel view to represent the structure of the molecule, only the van der Waals force is not enough to represent the structures of the molecule.

Therefore, a grid-based method is introduced to extract the molecular features and study the nature of toxicity in this paper. Multi-channel data is utilized to improve the prediction performance. It can be seen from the experimental results that the proposed method is more accurate and robust. The contributions of the method are listed as follows:

- A two-dimensional grid approach for toxicity prediction of the molecule is proposed.
- In order to improve the accuracy of toxicity prediction, Van der Waals forces and hydrogen bonding properties are combined to improve generalization performance.
- The grid calculation of the constructed molecules reveals the contribution of each atom of the molecule to the prediction. The different grid weight values are calculated by this method, indicating that atoms with higher grid weight values correspond to higher contributions in prediction.

## 2. Methods

### 2.1. Dataset

The Tox21 [14,30] dataset is currently the most widely used toxicity research database in the world. The molecules in the Tox21 database could obtain the exact values of corresponding properties through experimental detection, which could be used to verify our method.

There are 12 toxic substances in Tox21 are shown in Table 1, including the stress response effects (SR) and the nuclear receptor effects (NR). The SR includes five types (ARE, HSE, ATAD5, MMP, p53), and NR includes seven types (ER-LBD, ER, Aromatase, AhR, AR, AR-LBD, PPAR). Regarding the property ER of NR, Y Asako et.al used the high-performance prediction based on chemical structures [36]. Both the SR and NR effects are closely related to human health. For example, the activation of nuclear receptors can disrupt endocrine system function [37], and the activation of stress response pathways can lead to liver damage or cancer [38]. The Tox21 database contains the results of high-throughput screening for these 12 toxic effects. Based on this database, a corresponding grid-based deep learning model was constructed in this paper to predict whether a certain property of a molecule was toxic or not.

### 2.2. Method Overview

**Figure 1:**
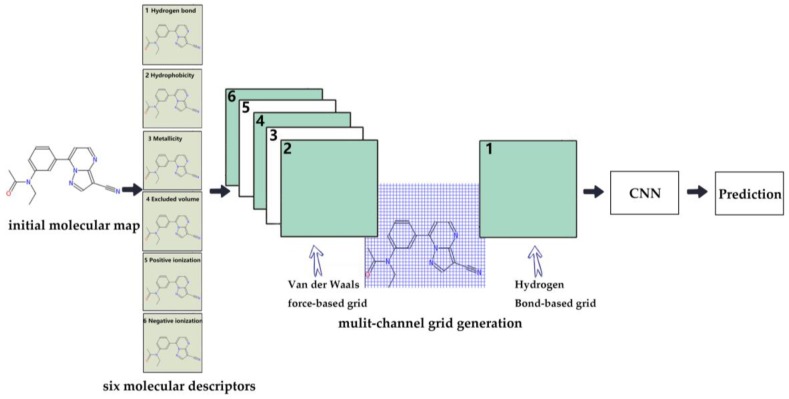
The work flow of the proposed method.

The whole process of the proposed structure is described as follow. First, the appropriate molecular descriptors are selected and extracted. These are beneficial for prediction. Secondly, the molecular grid is constructed to obtain the corresponding data according to the molecular descriptor. Then, the data are fed into the convolution neural network to obtain the prediction results. Moreover, in order to solve the problem of the unbalanced distribution of the positive and negative data sets in the Tox21 database, the method of data enhancement is introduced. The flow chart of this method is shown in Figure 1.

### 2.3. Construction of Molecular Descriptors

An important step in predicting the toxicity of a molecule is to construct suitable molecular descriptors. Moreover, in order to ensure that the descriptors could be fed into a deep learning network, the size of them should be fixed for all molecules. Therefore, six multi-channel grid descriptors were introduced in this paper. Based on the molecular grid descriptors [35] which have been used for predicting binding sites of proteins, the proposed grid descriptors in this paper were especially designed and suitable for molecular toxicity.

There are many properties of a single molecule or between multi-molecules used for property prediction, such as hydrophobicity, hydrogen bonding, ionization and metallicity. In the authors’ opinion, more properties could be helpful in distinguishing different activities of molecules. In order to obtain the property information of the molecule more precisely, six descriptors were introduced. These are shown in Table 2.

**Figure 2:**
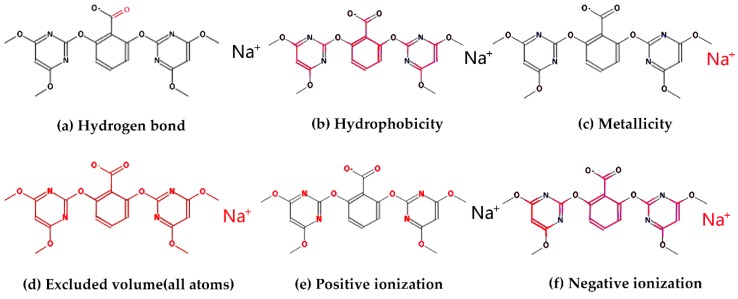
Multi-channel molecular descriptors. (**a**) the descriptor of Hydrogen bond, (**b**) the descriptor of Hydrophobicity, (**c**) the descriptor of Metallicity, (**d**) the descriptor of Excluded volume, (**e**) the descriptor of Positive ionization, (**f**) the descriptor of Negative ionization.

Based on the above description of molecular descriptors and the characteristics of the atoms contained in the data, each molecule could be divided into multi-channel data. For each descriptor, only related atoms are left in the channel. A case of molecule multi-channel descriptors is shown in Figure 2. A molecule in the NR-AhR property was selected. The corresponding atoms and bonds are highlighted in each channel.

### 2.4. Construction of Molecular Grid

All channels extracted from the molecules are not suitable to input a learning network directly. Therefore, six 2D grids were constructed over the channels respectively. Moreover, in order to deep discover the structure and property information of a molecule and ease the sparse problem, two calculation methods were introduced to calculate and generate grids with respect to different channels.

The Van der Waals force-based grid: The Van der Waals force is important to the molecular property, where all atom types are involved. The contribution of the Van der Waals force from each atom of each grid pixel is calculated through Equation (1)

(1) $$W{\left(g\right)}_{vdw}=\sum_{i=1}^{N}\left(1-\exp(-\left(\frac{r_{vdw}\left(a_{i}\right)}{r_{a}\left(g,a_{i}\right)}{)}^{12}\right)\right)$$

where $a_{i}$ represents the i*th* atom of a molecule, and *r_vdw_*(*a*) represents the van der Waals force radius of each type of atom. *r_a_*(*g,a*) represents the distance of each atom a from the specified grid pixel *g*. *W*(*g*)*_vdw_* represents the sum of the attribute values of pixel *g* from all atoms of within the specified range. The Van der Waals force was applied on the first five properties in Table 2 to generate grids. 

The hydrogen bond-based grid: The information of the hydrogen bond donor and the corresponding atom of the hydrogen bond acceptor were utilized to generate another grid through Equation (2), which was applied on the last property of Table 2.

(2) $$W{\left(g\right)}_{hbond}=\sum_{i=1}^{N}\left(\left(\frac{C}{r{\left(g,a_{i}\right)}^{12}}\right)-\frac{D}{r{\left(g,a_{i}\right)}^{10}}\right))$$

where *W*(*g*)*_hbond_* represents the hydrogen bonds of all atoms in each grid pixel *g*. *a_i_* represents each atom, *r*(*g,a*) represents the Euclidean distance of each atom to each grid pixel point. *C* is the well depth parameter assigned according to the hydrogen bonds with oxygen and nitrogen, and *D* is the well depth parameter assigned according to the hydrogen bonds with sulfur. The hydrogen bond is calculated through Autodock [39].

**Figure 3:**
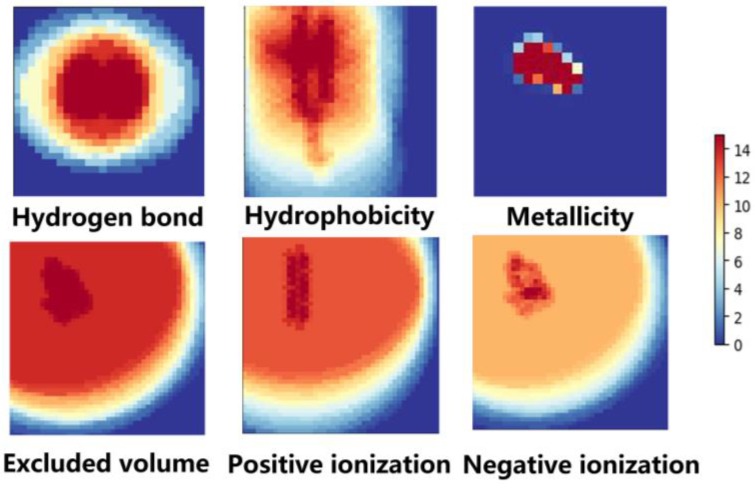
Heat map of six descriptors. The distribution of a two-dimensional grid of the five channels (positive/negative ionization, excluded volume, metallicity and hydrophobicity) were calculated using Van der Waals force-based grid method, and one channel about the hydrogen bond used the hydrogen bond-based grid method.

For each grid, it is generated according to its corresponding method. These 2D grids descriptors for each molecule are more clear and more specific. The final grid construction process is shown in Figure 3. The grid size is 24Å × 24Å, and the resolution of grid was set as 0.5Å × 0.5Å. In the experiment section, the performance of different resolutions were tested and displayed. The performance of 0.5Å × 0.5Å is the best among them. With the assistance of the grid, the structure and chemical information of each molecule were extracted. This can be fed into a convolutional neural network for training.

### 2.5. Convolutional Neural Network Architecture

The deep neural network, especially the convolutional neural network, is a feedforward neural network whose artificial neural unit can respond to a surrounding unit in a part of the coverage [40]. A convolutional neural network consists of one or more convolutional layers and a fully connected layer (corresponding to a classical neural network). Which is helpful for a two-dimensional structure as the input data. Compared with the other depth and feedforward neural networks, convolutional neural networks consider fewer parameters, which is an attractive deep learning structure.

**Figure 4:**
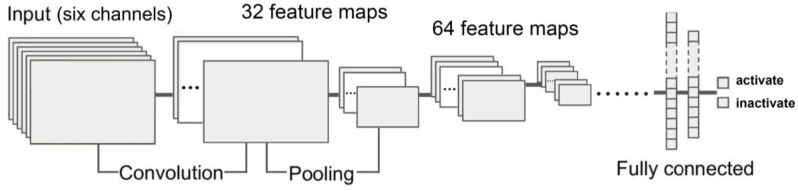
The structure of convolutional neural network (CNN).

In this paper, the convolutional neural network was used to predict the toxicity of the molecule and identify the key functional part of the molecule. The reason why the authors choose a simple version CNN is that the calculated parameters are relatively large for some complicated networks. Reducing the number of network layers could ensure accuracy and prevent over-fitting [41]. Through the experiment, this study found that four layers of the structure is the optimal selection to obtain the best results. Figure 4 shows the structure of the convolutional neural network.

During the input process, each molecule is described as a multi-channel specific two-dimensional array. Both the input layer and the hidden layer use the same activation function f.

Loss Function: The loss function is used to estimate the degree of inconsistency between the predicted value y(p) of the model and the true value Y, which is a binary classification in the toxicity classification. The binary cross entropy is selected as the loss function which are defined as follows:

(3) $$loss=-\sum_{i=1}^{n}[\left({\hat{y}}_{i}\log y_{i}+\left(1-{\hat{y}}_{i}\right)\log(1-{\hat{y}}_{i}\right)]$$

where *n* represents the number of categories of the classification, and ${\hat{y}}_{i}$ represents the label. For a binary classification, ${\hat{y}}_{i}$ represents 0 or 1. $y_{i}$ represents the corresponding probability of the label.

In addition, the advantage of the CNN is that different layers of the structure reveal the significance of a molecule. Therefore, the largest and most concentrated values of the feature map of each layer could be regarded as the important positions of the molecule.

**Figure 5:**
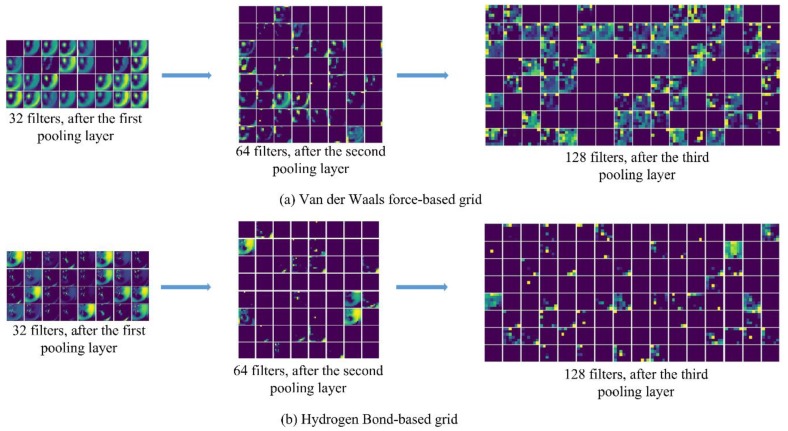
Visualization of training process. Three feature maps output after each pooling layer with different filters are displayed. The part (**a**) is grid from excluded volume, and the part (**b**) is grid from Hydrogen Bond.

The six descriptors were calculated by two equations in Section 2.4, and the data of six channels were obtained and sent to the convolution neural network for training, and the feature map of the output data of each layer was obtained. The results of each layer after processing by the CNN are shown in Figure 5. The top part of the figure is the grid from the excluded volume, the first one is the visualization output through 32 filters of a two-dimensional convolution network structure, and the second and third are the results through 64 filters and 128 filters, as shown in Figure 5a. The second part of the figure is the grid from the hydrogen bond. Similarly, the feature maps of the output from each layer are shown in Figure 5b.

### 2.6. Data Augmentation

Due to the imbalance between the active and inactive data in the Tox21 dataset, data augmentation was implemented in the training process. There are 12 properties in Tox21 indicating whether a property is active or inactive of a molecule. However, not all molecules record all 12 properties performances, and the distribution of positive and negative samples of some properties are uneven which can affect the performance of the training network. For example, the distribution of positive and negative samples of NR-AR-LBD and SR-ATAD5 is very unbalanced, such as, in the training data of AR-LBD, the positive-negative ratio is 236:7798, that is, the number of negative samples is approximately 33 times that of the positive samples. Therefore, the synthetic minority oversampling technique (SMOTE) [42] was introduced in this paper. This is an up-sampling method for data processing of the unbalanced data. The core of the method is to generate more samples using existing samples. SMOTE is the most common method, which uses the interpolation between a small number of samples to generate additional samples. Moreover, the weights of positive samples and one-hot coding are also increased to solve the unbalance problem.

## 3. Experiment

### 3.1. Data Processing

The introduction of the training set, test dataset and verification set are shown in Table 3. In the table, the number of active samples, the number of total samples and the proportion of positive samples in the training sets, test set and verification set are displayed. Through the previous introduction, the problem of the dataset has already been discussed. It is obvious that the distributions of the active dataset and inactive dataset of 12 properties are uneven.

Moreover, the data enhancement methods were used in the training data, and batch normalization [43] and dropout [44] were also introduced to prevent the data from over-fitting.

Before the features of the data were extracted, all data were standardized and cleaned. First, the Standardizer software (version 19.18.0, ChemAxon, Budapest, Hungary) was used for standardization. Each molecule was added explicit hydrogens and aromatized. Then, the duplicated data were cleaned according to InChIKeys values [45]. The rule for deleting molecules is that for different molecules with the same InChIKeys, these molecules are deleted when they have different values for the same property. 

### 3.2. Results of Different Layers in Convolutional Networks

**Figure 6:**
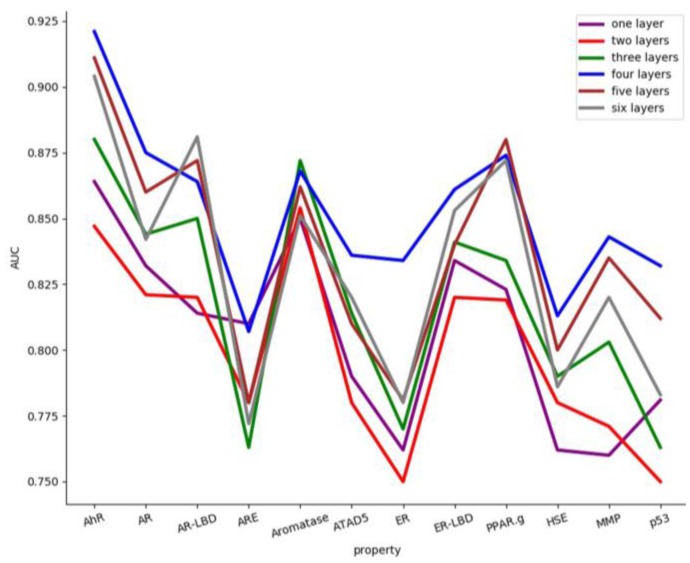
The area under the curve (AUC) of 12 properties with different hidden layers. The different colors represent 12 AUC values with different hidden layers.

Through the introduction of the CNN, we designed four hidden layers CNN were designed. In order to select the most appropriate number of layers, a comparative experiment with different layers was implemented. The structure of the convolution neural network was chosen with more than four layers (five layers and six layers) and less than four layers (one layer, two layers and three layers) for comparison. The area under curve (AUC) results indicate that there are approximately 12 properties as shown in Figure 6.

It is obvious that when the number of hidden layers is set to 4, the best result is obtained. It is possible that when the hidden layers are relatively small, the network may not be trained well and the network performance is very poor. If the number of hidden layers is too large, the network system error can be reduced, and the network training time can be prolonged. Moreover, the training process can easily fall into the local minimum and cannot find the optimal point.

### 3.3. Hyperparameter and Loss

All 12 toxic properties can be predicted by the proposed model. For the different properties, there are some parameter adjustments during the test, as shown in Table 4. 

From the data in Table 4, it can be seen that the hyperparameter settings of each property have different choices. The setting of the hyperparameter for the training process of each property are also provided in Appendix A.

**Figure 7:**
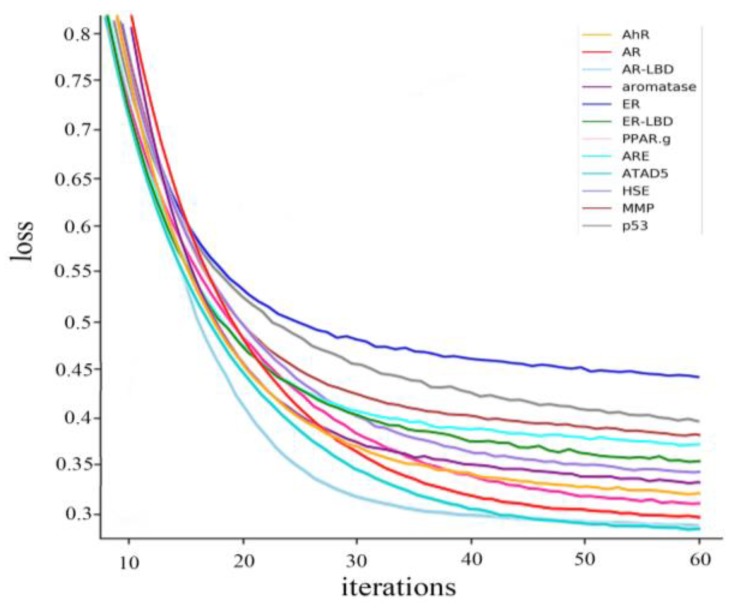
Training loss curve.

The loss during the training process was also recorded. In order to pay more attention to the changes of loss, the loss curves for each property are shown in Figure 7.

According to the 60 iterations of training in Figure 7, the loss curves of each property show a downward trend and become convergent after 30 iterations.

### 3.4. Evaluation on Different Channels

**Figure 8:**
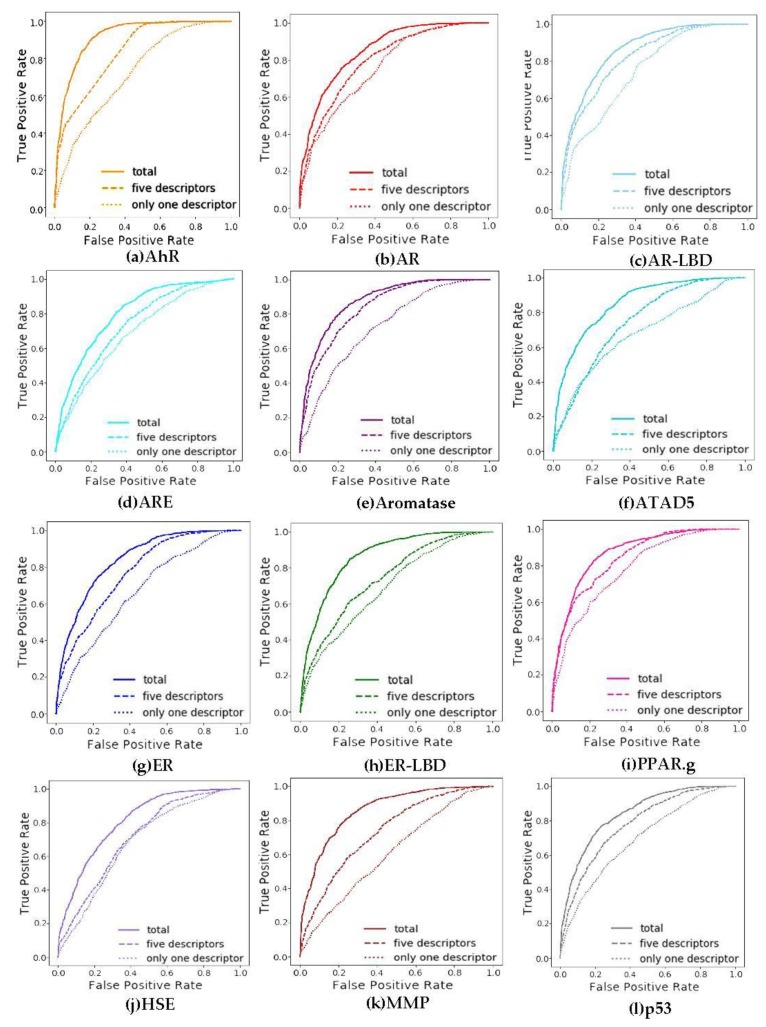
The receiver operating characteristic (ROC) curves of the 12 properties. (**a**) ROC curves of AhR, (**b**) ROC curves of AR, (**c**) ROC curves of AR-LBD, (**d**) ROC curves of ARE, (**e**) ROC curves of Aromatase, (**f**) ROC curves of ATAD5, (**g**) ROC curves of ER, (**h**) ROC curves of ER-LBD, (**i**) ROC curves of PPAR.g, (**j**) ROC curves of HSE, (**k**) ROC curves of MMP, (**l**) ROC curves of p53.

However, this study also realized the importance of extracting features. Two methods were used in the feature extraction part, the first one was used to extract the van der Waals force and the second one was used to calculate the hydrogen bond. It is important to demonstrate that combining two girds provides a better way to explicate the nature of a molecule. Therefore, the comparisons between the different grid methods including only each one of the grid calculation methods and the combination of both methods are show in Figure 8. It was demonstrated that the performance with two methods fusion is better than any one of them. It is possible that the combined results of the two methods are most suitable for feature extraction which reveal the nature of toxicity.

Each property contains three receiver operating characteristic (ROC) curves, in which the solid line represents the result of the calculation of the six descriptors, the dashed line represents the result after the calculation of the five descriptors by Van der Waals force, and the dotted line represents the result after the calculation of a descriptor refined the hydrogen bond.

**Figure 9:**
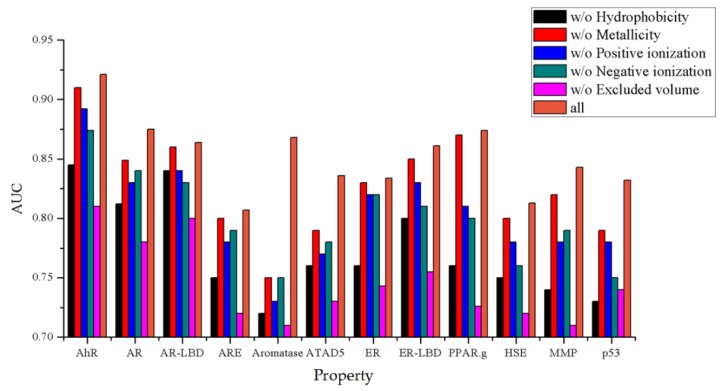
The results of combining all descriptors (brown) and removing one of the five descriptors.

From the comparison of ROC curves in Figure 8, it can be seen that the best choice is to extract the properties of six channels. In addition, the Van der Waals force equation was used to calculate the five descriptors, and the hydrogen bond equation was used to calculate the other descriptor. Compared with the two methods, the former has a greater positive impact on the results. In order to explore which of the five channels using the Van der Waals force method has the most significant impact on the results, this study adopted a comparative experiment. Any four channels of the Van der Waals force method were used and combined with hydrogen bonds for comparison. The experimental results are shown in Figure 9. From Figure 9, it is obvious that the properties excluded the volume and hydrophobicity which have great influence on the results, and combining all the descriptors together obtains the best results.

### 3.5. Evaluation on Different Resolutions

**Figure 10:**
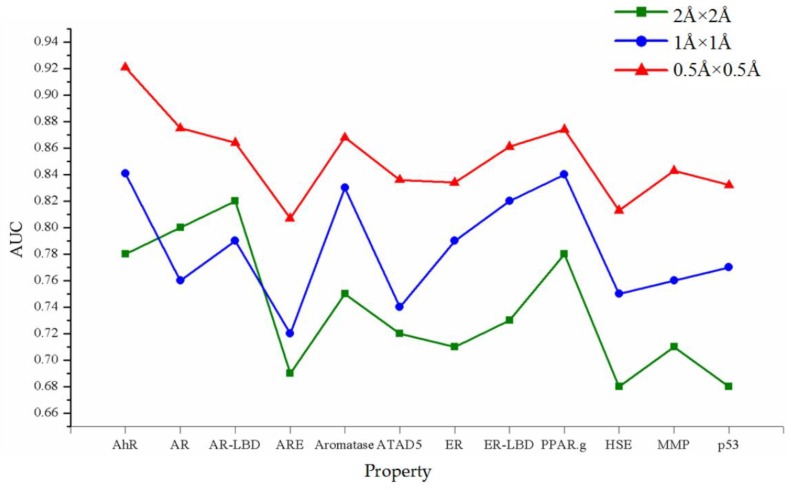
The results of the different resolutions. The red line represents the result of the resolution of 0.5Å × 0.5Å, while the yellow and green lines represent 1Å × 1Å and 2Å × 2Å respectively.

Moreover, the resolution is also important for prediction accuracy. Three resolutions were tested including 0.5Å × 0.5Å, 1Å × 1Å and 2Å × 2Å. The results of different resolutions are shown in Figure 10. It can be seen from Figure 10 that as the resolution value becomes higher, the performance of prediction becomes more accurate. When the resolution is 0.5Å × 0.5Å, the average AUC value of 12 properties is 0.84, and when the resolution is 1Å × 1Å and 2Å × 2Å, the average AUC values are 0.81 and 0.76 respectively. Therefore, the resolution of 0.5Å × 0.5Å is the most suitable for model training.

### 3.6. Comparison of Different Methods

In this section, the most common algorithms and their reported results have been introduced for comparison, including RF, SVM, multi-task learning, and several deep learning methods that appeared in the Tox21 Challenge. A random forest is a classifier that contains multiple decision trees, and the output category is determined by the mode of the category output by the individual tree [41]. The SVM is a generalized linear classifier that performs binary classification on data in supervised learning. As most of the molecules in the dataset are labeled with multiple toxic properties, a multitask classifier can be applied for prediction which is implemented in DeepChem [46]. Chemception developed the convolutional neural networks [31] to predict toxicity using 2D images of compounds without explicitly calculating chemical descriptors and achieved an average AUC 0.77. Mykola Galushka et al. utilized a pre-trained auto-encoder [32] to build classifiers to predict the toxicity property. The AMAZIZ team used consensus modeling for predictions based on the ASNN [33]. Capuzzi et al. used the DNN [34] with molecular descriptors and achieved an average AUC of 0.83 for both 12 properties. The comparison and results are summarized in Table 5. 

It could be seen from Table 5 that the proposed multi-channel grid CNN method performs best among all other methods overall. For properties of AhR, AR, ARE, Aromatase, ER, ER-LBD, PPAR.g and HSE, our method did get the first place. The average AUC values of each method were compared. Our method is approximately 0.8, 0.7, 0.9, and 0.2 higher than Chemception, Auto-encoder, ASNN, DNN methods respectively, which are also based on deep learning methods. The AUC value of the proposed method is also 0.6 higher than that of the random forest method.

## 4. Conclusions

In daily life, people are exposed to many molecules every day through food, drugs, etc. Therefore, the toxicity test of a molecule is crucial to human life. Through the Tox21 database, the nature of the 12 toxic substances can be studied which is very meaningful work. Previously, the traditional machine learning method was not considered to discover the nature of molecular data. The method proposed in this paper extracted the descriptors of the atomic type, distance, coordinate position and other information according to the molecular characteristics, and a multi-channel grid-based CNN was constructed for toxicity prediction. The result shows that the proposed method outperforms other convolution machine learning methods and deep learning methods.

However, our method is to extract the properties of the 2D grid created by the molecule. The study of the properties of a grid-based molecule can continue to be studied through other perspectives. In future research, the authors intend to continue to expand the depth and breadth of research based on the current direction, such as introducing more accurate molecular descriptors and the grid generation method. On the other hand, other effective and fast methods of deep learning can be found which could improve the accuracy of the prediction. 

## Figures and Tables

**Table 1:** Content of 12 properties. The left side are the seven toxic properties of the nuclear receptor effects (NR), and the right side are the five toxic properties of the heat stress response effects (SR).

Nuclear Receptor Panel		Stress Response Panel	
ER-LBD	estrogen receptor alpha, luciferase	HSE	heat shock factor response element
ER	estrogen receptor alpha	ATAD5	genotoxicity indicated by ATAD5
AhR	aryl hydrocarbon receptor	MMP	mitochondrial membrane potential
AR	androgen receptor	p53	DNA damage p53 pathway
PPAR.g	peroxisome proliferator-activated receptor gamma	ARE	nuclear factor (erythroid-derived 2)-like 2 antioxidant responsive element
AR-LBD	androgen receptor, luciferase		
Aromatase	cytochrome P450 enzymes		

**Table 2:** The molecular descriptors.

Channel	Descriptor
1	Hydrogen bond
2	Hydrophobicity
3	Metallicity
4	Excluded volume
5	Positive ionization
6	Negative ionization

**Table 3:** Data distribution. The table shows the size of the training data set and test data set for each property, as well as the distribution of validation data set for each property.

	Train Active/Total	Test Active/Total	Verification Active/Total
AhR	776/7404 (10.48%)	73/600 (12.17%)	31/274 (11.31%)
AR	285/8472 (3.36%)	12/576 (2.08%)	3/294 (1.57%)
AR-LBD	236/7798 (3.03%)	8/574 (1.39%)	4/255 (1.19%)
ARE	940/6524 (14.41%)	93/546 (17.03%)	48/236 (20.34%)
Aromatase	298/6621 (4.50%)	36/519 (6.94%)	18/216 (8.33%)
ATAD5	265/8228 (3.22%)	38/612 (6.21%)	25/274 (9.12%)
ER	716/6967 (10.28%)	49/509 (9.63%)	27/267 (10.11%)
ER-LBD	343/7960 (4.31%)	19/589 (3.23%)	10/289 (3.46%)
PPAR.g	192/7432 (2.58%)	31/595 (5.21%)	15/269 (5.58%)
HSE	348/7398 (4.70%)	22/601 (3.66%)	10/269 (3.71%)
MMP	966/6619 (14.59%)	60/536 (11.19%)	38/240 (15.83%)
p53	447/7828 (5.71%)	40/605 (6.61%)	28/271 (10.33%)

**Table 4:** Hyperparameter settings of the neural networks.

Hyperparameter	Values are Selected
number of hidden units	64, 128, 256, 512, 1024, 2048
numbers of hidden layers	1, 2, 3, 4, 5, 6
learning rate	$10^{-3}$, $10^{-4}$, $10^{-5}$
dropout rate	20% input layer et.al
L2 weight decay	$10^{-2}$, $10^{-3}$

**Table 5:** AUC results of different methods. In the methods listed in the table, the best value of each property is marked in bold. The results of all other methods except ours are obtained through their publications.

	Traditional Machine Learning			Deep Learning				
	SVM	Multitask	RF	Chemception	Auto-Encoder(MLP)	ASNN	DNN	Our Method
**AhR**	0.823	0.853	0.892	0.80	0.84	0.853	0.90	**0.921**
**AR**	0.643	0.734	0.718	0.757	0.81	0.736	0.83	**0.875**
**AR-LBD**	0.832	0.844	0.740	0.886	0.85	0.650	**0.89**	0.864
**ARE**	0.768	0.792	0.785	0.654	0.75	0.729	0.76	**0.807**
**Aromatase**	0.700	0.795	0.783	0.799	0.82	0.737	0.86	**0.868**
**ATAD5**	0.777	0.786	0.828	0.776	0.78	0.741	**0.85**	0.836
**ER**	0.615	0.637	0.769	0.694	0.74	0.749	0.74	**0.834**
**ER-LBD**	0.715	0.773	0.750	0.762	0.79	0.715	0.83	**0.861**
**PPAR.g**	0.644	0.741	0.777	0.819	0.69	0.785	0.70	**0.874**
**HSE**	0.704	0.700	0.775	0.717	0.70	0.799	0.79	**0.813**
**MMP**	0.830	0.822	0.926	0.755	0.85	0.904	**0.95**	0.843
**p53**	0.738	0.697	0.782	0.776	0.75	0.765	**0.84**	0.832
**average**	0.73	0.76	0.79	0.77	0.78	0.76	**0.83**	**0.85**

## Appendix A

**Table A1:** Hyperparameter settings of 12 properties.

	Learning Rate	Dropout Rate	L2 Weight Decay
**AhR**	0.00001	25% input layer	0.0001
**AR**	0.000001	20% input layer	0.0001
**AR-LBD**	0.000001	20% input layer	0.001
**ARE**	0.0001	20% input layer	0.0001
**Aromatase**	0.00001	20% input layer	0.0001
**ATAD5**	0.00001	--	0.0001
**ER**	0.00001	--	0.0001
**ER-LBD**	0.00001	--	0.001
**PPAR.g**	0.0001	--	0.0001
**HSE**	0.00001	--	0.001
**MMP**	0.0001	25% input layer	0.0001
**p53**	0.0001	--	0.001
